# A Model for Predicting Polycystic Ovary Syndrome Using Serum AMH, Menstrual Cycle Length, Body Mass Index and Serum Androstenedione in Chinese Reproductive Aged Population: A Retrospective Cohort Study

**DOI:** 10.3389/fendo.2022.821368

**Published:** 2022-03-17

**Authors:** Huiyu Xu, Guoshuang Feng, Kannan Alpadi, Yong Han, Rui Yang, Lixue Chen, Rong Li, Jie Qiao

**Affiliations:** ^1^ Center for Reproductive Medicine, Department of Obstetrics and Gynecology, Peking University Third Hospital, Beijing, China; ^2^ National Clinical Research Center for Obstetrics and Gynecology, Beijing, China; ^3^ Key Laboratory of Assisted Reproduction (Peking University), Ministry of Education, Beijing, China; ^4^ Beijing Key Laboratory of Reproductive Endocrinology and Assisted Reproductive Technology, Beijing, China; ^5^ Center for Clinical Epidemiology and Evidence-Based Medicine, Beijing Children’s Hospital, Capital Medical University, National Center for Children’s Health, Beijing, China; ^6^ The Predict Health, Houston, TX, United States; ^7^ Hangzhou Qingguo Medical Technology Co. Ltd., Hangzhou, China

**Keywords:** PCOS, website-based tool, AMH, menstrual cycle length, BMI, androstenedione

## Abstract

**Background:**

A clinical diagnosis of polycystic ovary syndrome (PCOS) can be tedious with many different required tests and examinations. Furthermore, women with PCOS have increased risks for several metabolic complications, which need long-term health management. Therefore, we attempted to establish an easily applicable model to identify such women at an early stage.

**Objective:**

To develop an easy-to-use tool for screening PCOS based on medical records from a large assisted reproductive technology (ART) center in China.

**Materials and Methods:**

A retrospective observational cohort from Peking University Third Hospital was used in the study. Least Absolute Shrinkage and Selection Operator (LASSO) logistic regression with 10-fold cross-validation was applied to construct the model. The area under the receiver operating characteristic curve (AUC), sensitivity, and specificity values were used to evaluate and compare the models.

**Design, Setting, and Participants:**

This retrospective cohort study included 21,219 ovarian stimulation cycle records from January to December 2019 in Peking University Third Hospital.

**Main Outcomes and Measures:**

The main outcome was whether there was a clinical diagnosis of PCOS. The independent variables included were age, body mass index (BMI), upper limit of menstrual cycle length (UML), basal serum levels of anti-Müllerian hormone (AMH), testosterone androstenedione, antral follicle counts et al.

**Results:**

We have established a new mathematical model for diagnosing PCOS using serum AMH and androstenedione levels, UML, and BMI, with AUC values of 0.855 (0.838–0.870), 0.848 (0.791–0.891), 0.846 (0.812–0.875) in the training, validation, and testing sets, respectively. The contribution of each predictor to this model were: AMH 41.2%; UML 35.2%; BMI 4.3%; and androstenedione 3.7%. The top 10 groups of women most predicted to develop PCOS were demonstrated. An online tool (http://121.43.113.123:8888/) has been developed to assist Chinese ART clinics.

**Conclusions:**

The models and online tool we established here might be helpful for screening and identifying women with undiagnosed PCOS in Asian populations and could assist in the long-term management of related metabolic disorders.

## Introduction

Polycystic ovary syndrome (PCOS) is one of the most common endocrine and metabolic disorders in women of reproductive age, with a prevalence of 5.6% in the Chinese population (1). Despite growing interest in understanding the pathophysiology of PCOS, our knowledge is still incomplete. The diagnosis of PCOS in assisted reproductive technology (ART) clinics can be challenging, and women with this disorder can have different signs and symptoms: some might only have a few, whereas others can experience many. Even within the same woman, the number of symptoms experienced and their severity will change over time. Therefore, the final diagnosis of PCOS can be long and tedious with many different required tests and examinations, which means that many women might not recognize that they have the PCOS associated conditions and not seek appropriate evaluation and treatment. Furthermore, screening and diagnosis of PCOS is not an easy task for primary physicians. These factors mean that PCOS is largely underdiagnosed. Indeed, a large retrospective study found that the prevalence of PCOS in women attending primary care ART clinics was lower than in community samples, indicating a general problem of underdiagnosis (2).

In addition to androgen excess, insulin resistance is indicated to play an important role in pathophysiology of PCOS (3–5). For a specific PCOS woman, the symptoms change over time, from hirsutism in adolescence to infertility in reproductive age. Even after menopause, high levels of androgens and insulin resistance continues, which results in higher risk of type 2 diabetes and other related metabolic syndrome (6, 7). Therefore, irregular menstruation and/or hirsutism can no longer be regarded as a benign nuisance. Long-term health managements, including tailored medication and lifestyle management have been paid more and more attention (4). The premise of giving PCOS women long-term health management is to early identify them. Here we attempted to establish an online tool for screening PCOS, which might be helpful for timely identification of the undiagnosed PCOS and thus improve long-term health management for affected women.

## Materials and Methods

### Subjects

**Figure 1:**
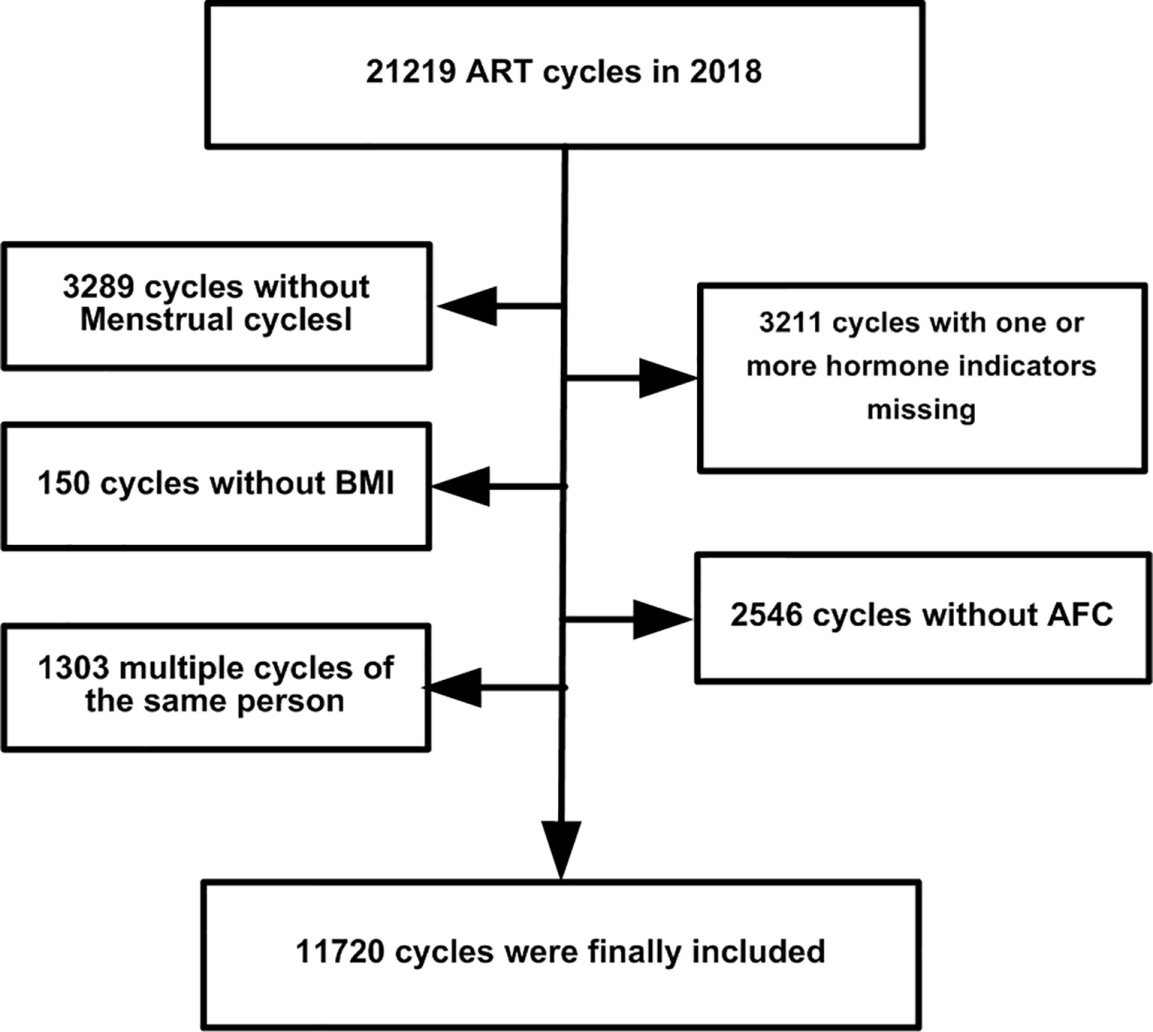
The design of this study.

This was a retrospective observational cohort study in Peking University Third Hospital. In all, 21,219 ovarian stimulation cycle records were recovered from January to December 2019. We excluded the following: 3,289 cycles without menstrual cycle data; 150 without body mass index (BMI) information; 3,211 without one or more key hormone levels recorded; 2,546 without information on the antral follicle count (AFC); and 1303 multiple cycles of the same patient. The study flowchart is shown in **Figure 1**. Finally, 11,720 cycle records were used. The menstrual cycle duration in this study refers to the upper limit of menstrual cycle length (UML). For example, if the duration of a patient’s menstrual cycle was 30–90 days, 90 days was used as the UML. The basic characteristics of the cycle data are listed in **Table 1**. The need for informed consent by the patients was waived for the deidentified data in our analysis, which conformed to the Helsinki declaration (8).

**Table 1:** Basic characteristics.

	PCOS (*n* = 1225)	non-PCOS (*n* = 10495)
Age (years)	31.4 ± 5.0	33.6 ± 5.2
BMI (kg/m^2^)	24.1 (21.5–26.8)	22.2 (20.3–24.5)
UML (days)	40 (30–62.5)	29 (28–30)
AMH (ng/mL)	6.4 (3.5–10.1)	2.1 (1.0–3.8)
FSH (IU/L)	5.9 (4.7–7.2)	6.7 (5.2–8.4)
LH (IU/L)	4.3 (2.7–6.7)	3.4 (2.2–4.7)
E_2_ (pmol/L)	174 (135–220)	161 (122–209)
TES (nmol/L)	0.7 (0.7–1.1)	0.7 (0.7–0.7)
AND (nmol/L)	8.9 ± 4.9	5.8 ± 3.1
AFC	10 (6–14)	10 (7–15)

BMI, body mass index; UML, the upper limit of menstrual cycle length; AMH, anti-Müllerian hormone; FSH, follicle-stimulating hormone; LH, luteinizing hormone; E_2_, estradiol; TES, testosterone; AND, androstenedione; AFC, antral follicle count.

### Diagnosis of PCOS

Women with PCOS were diagnosed according to the 2003 Rotterdam criteria (9), which require the presence of at least any two of the following: (1) ovulatory dysfunction (i.e., oligo/anovulation); (2) hyperandrogenism, diagnosed either clinically by cutaneous manifestations of androgen excess or hyperandrogenemia (high testosterone or androstenedione levels in blood tests); or (3) polycystic ovaries on ultrasonography. Diagnoses of PCOS were made after the exclusion of phenotypically similar androgen excess disorders such as congenital adrenal hyperplasia, androgen-secreting tumors, Cushing syndrome, thyroid dysfunction, or hyperprolactinemia.

Hyperandrogenism was diagnosed by the presence of excessive acne, androgenic alopecia, or hirsutism; or chemically by high serum levels of total testosterone (TES) ≥ 2.5 nmol/L or androstenedione (AND) ≥ 11.5 nmol/L. Hirsutism was diagnosed as described (10). Briefly, a modified Ferriman–Galwey score of >4, or hair growth involving the upper lip, thighs, and lower abdomen with scores of >2 are used to diagnose hirsutism in our clinical practice. Oligomenorrhea was diagnosed as menstrual cycles lasting >35 days but <6 months. Amenorrhea was defined as the absence of menstruation for more than 6 months after a cyclic pattern had been established. A polycystic ovary on ultrasonography was defined as one containing 12 or more follicles measuring 2–9 mm in diameter or an ovary with a volume of greater than 10 mL. A single ovary meeting either or both of these definitions was deemed sufficient for the diagnosis. Hyperprolactinemia was diagnosed from two serum prolactin levels of >25 ng/mL. The clinical diagnosis of having or not having PCOS was recorded by our data supporting group, along with other diagnoses and basal or clinical information.

### Antral Follicle Counts and Endocrine Assays

Antral follicles measuring 2–10 mm in diameter in both ovaries were counted on menstrual cycle day 2 using transvaginal ultrasound scans. On the same day, intravenous blood was collected for measuring follicle stimulating hormone (FSH), luteinizing hormone (LH), TES, AND, and estradiol (E_2_) concentrations. Blood samples for measuring AMH were collected on any day of the menstrual cycle prior to ovarian stimulation. Samples were collected, immediately inverted five times and centrifuged at 1800g 10min for further endocrine assessments.

Serum levels of FSH, LH, TES, AND, and E_2_ measurements were tested using a Siemens Immulite 2000 immunoassay system (Siemens Healthcare Diagnostics, Shanghai, P. R. China). The quality controls for FSH, LH, TES, AND, and E_2_ were supplied by Bio-RAD Laboratories (Hercules, CA, USA; Lyphochek Immunoassay Plus Control, Trilevel, catalog number 370, lot number 40370). Serum AMH concentrations were measured using an ultrasensitive two-site enzyme-linked immunosorbent (ELISA) assay (Ansh Laboratories LLC; Webster, TX, USA), using quality controls supplied with the ELISA kits. The coefficients of variation for the assays were <6% for AMH, FSH, and LH, and <10% for E_2_, AND, and TES.

### Statistical Analysis

The diagnosis of having or not having PCOS was included as the dependent variable, and AFC, AMH level, age and other measures were included as independent variables. To make the model better applied to the clinical practice, continuous variables were transformed into categorical variables. The grouping standard for independent variables was mainly based on data exploration before analysis combined with our clinical experience. The grouping criteria for each independent variable in the three different models were held the same. For variable selection process, first, a proportion (70%) of the data was selected randomly as a training set, which was used for model establishment, and the rest (30%) of the data was used as the testing set, which was used for model evaluation. Then a prediction model was constructed in the training set. The scaled negative loglikelihood (–*Log* L *(β)*) was used to evaluate the final model: the smaller the value of scaled –*Log* L (*β*) in the validation set the better the model fit. The logistic Least Absolute Shrinkage and Selection Operator (LASSO) model, a shrinkage method that can actively select from a large and potentially multicollinear set of variables and reduce the likelihood of overfitting, was applied to construct a predictive model. The logistic LASSO is a logistic regression analysis approach that penalizes the absolute size of the coefficients of a regression model based on the value of penalty term, λ. With larger penalties, the estimates of weaker factors shrink toward zero, so that only the strongest predictors remain in the model. The value of λ was determined with 10-fold cross validation, and the most predictive covariates—selected by the minimum value (λ min)—were used to construct the PCOS diagnostic models (11). The performance of each model was assessed using the area under the receiver operator characteristic curve (AUC), sensitivity and specificity with 95% confidence interval (CI). All the analyses in this study were performed using SAS JMP Pro (version 14.2; SAS Institute, Cary, NC, USA), and *p* < 0.05 was considered statistically significant.

## Results

The indicators in **Table 1** were all of significance in univariate analysis when diagnosing PCOS, and then included for multiple logistic regression with 10 fold cross-validation analysis. When seven variables were included, the scaled -*Log* L (*β*) value in the validation set no longer decreased indicated in model building process of Model1 in **Supplementary Document 1**, thus Model 1 was identified. The parameter estimations of Model 1 were shown in **Table 2**. The AMH level is generally considered to be a good substitute for the AFC for PCOS diagnosis (12–14), thus we tried to establish another model without AFC. All the independent variables except AFC were included to identify the ideal model, using the same multiple logistic regression method with cross validation, Model 2 were identified, the variables included were age, AMH, UML, BMI, TES and AND. The parameter estimations were indicated in **Supplementary Table 1**.

**Table 2:** Multiple analysis of the effects of each predictive variable on PCOS in Model 1 with AFC.

Variables	Parameter estimation (95% CI)	Standard error	Wald χ^2^	*p* value
Age [(35–40) vs <30]	0.0385 (-0.1625 - 0.2395)	0.1026	0.1407	0.7076
Age [(30–35) vs <30]	0.1513 (-0.0271 - 0.3298)	0.0910	2.7636	0.0964
UML [>90 vs ≤35]	-2.0530 (-2.3706 - -1.7353)	0.1621	160.4179	<.0001
UML [(60–90) vs ≤35]	-1.8190 (-2.1520 - -1.4860)	0.1699	114.6152	<.0001
UML [(45–60) vs ≤35]	-1.7885 (-2.0209- -1.5562)	0.1185	227.6347	<.0001
UML [(35–45) vs ≤35]	-1.4368(-1.6570 - -1.2166)	0.1123	163.5814	<.0001
BMI [≥28 vs <18.5]	-1.1120 (-1.5015 - -0.7225)	0.1987	31.3122	<.0001
BMI [(24–28) vs <18.5]	-0.7747 (-1.1296 - -0.4198)	0.1811	18.3078	<.0001
BMI [(18.5–24) vs <18.5]	-0.2294 (-0.5683- 0.1096)	0.1729	1.7593	0.1847
AMH [≥10 vs <2.5]	-1.6698 (-2.0006- -1.3389)	0.1688	97.8673	<.0001
AMH [(7.5–10) vs <2.5]	-1.3811 (-1.6817- -1.0805)	0.1534	81.0879	<.0001
AMH [(5–7.5) vs <2.5]	-0.9501 (-1.2046- -0.6956)	0.1299	53.5293	<.0001
AMH [(2.5–5) vs <2.5]	-0.2739 (-0.4934- -0.0545)	0.1120	5.9857	0.0144
TES [≥1.3 vs <0.7]	-0.1665 (-0.4591- 0.1261)	0.1493	1.2440	0.2647
TES [(1.1–1.3) vs <0.7]	0.1647 (-0.2162- 0.5456)	0.1943	0.7179	0.3968
TES [(0.9–1.1) vs <0.7]	-0.2113 (-0.5088- 0.0862)	0.1518	1.9382	0.1639
TES [(0.7–0.9) vs <0.7]	0.1803 (-0.0920-0.4527)	0.1389	1.6847	0.1943
AND [≥10 vs <5]	-0.6853 (-0.9428- -0.4278)	0.1314	27.2007	<.0001
AND [(5–10) vs <5]	-0.3156 (-0.5048- -0.1265)	0.0965	10.6972	0.0011
AFC [≥20 vs <10]	-1.6230 (-1.8891- -1.3569)	0.1358	142.9331	<.0001
AFC [(15–20) vs <10]	-0.8107 (-1.0534- -0.5680)	0.1238	42.8657	<.0001
AFC [(10–15) vs <10]	-0.2285 (-0.4377- -0.0194)	0.1067	4.5850	0.0323

The AUCs of Model 2 (without the AFC) in the training and validation data were 0.862 and 0.865, respectively, whereas the AUCs of Model 1 (with the AFC) in the training and validation data were 0.865 and 0.845, respectively. Inclusion of the AFC did not improve the performance of our models. The contribution of each variable is shown in **Table 3**. The main effect of AMH in Model 2 (without the AFC) was 35.1%, whereas the main effects of AMH and AFC in Model 1 (with the AFC) were 18.3% and 17.2%, respectively. Given the small contributions of Tes and age in Model 2 (without the AFC) (**Table 3**), we eliminated them in subsequent model building. UML, AMH, BMI, and AND levels were included in this process to derive Model 3. The estimated parameter values and *p* values of each index are shown in **Table 4**. The main effects of each predictor contributing to Model 3 were: AMH 41.2%; UML 35.2%; BMI 4.3%; and AND 3.7%. **Table 5** shows the AUC, sensitivity, and specificity of Model 3 in the training, validation and testing sets.

**Table 3:** Comparison of the main effects of each variable in Models 1 and 2.

Variables	Model 2 without AFC	Model 1 with AFC
UML	39.60%	39.40%
AMH	35.10%	18.30%
AFC		17.20%
BMI	3.40%	5.60%
AND	1.70%	1.50%
TES	0.30%	0.40%
Age	0.20%	0.20%

**Table 4:** Multiple analysis of the effects of each predictive variable on PCOS in Model 3.

Variables	Parameter estimation (95% CI)	Standard error	Wald χ^2^	*p* value
UML [≥90 vs (0,35)]	2.2259 (1.8810–2.5708)	0.176	159.9937	<.0001
UML [(60,90) vs (0,35)]	2.0083 (1.6498–2.3669)	0.1829	120.536	<.0001
UML [(45,60) vs (0,35)]	1.8418 (1.5889–2.0947)	0.129	203.742	<.0001
UML [(35,45) vs (0,35)]	1.4078 (1.1669–1.6486)	0.1229	131.2673	<.0001
BMI [≥28 vs (0,18.5)]	1.2152 (0.7922–1.6382)	0.2158	31.7069	<.0001
BMI [(24,28) vs (0,18.5)]	0.7843 (0.3958–1.1728)	0.1982	15.6543	<.0001
BMI [(18.5,24] vs (0,18.5)]	0.2845 (–0.0860–0.6551)	0.189	2.2657	0.1323
AMH [≥10 vs (0,2.5)]	2.5009 (2.1936–2.8082)	0.1568	254.4008	<.0001
AMH [(7.5,10) vs (0,2.5)]	2.1890 (1.8891–2.4888)	0.153	204.784	<.0001
AMH [(5,7.5) vs (0,2.5)]	1.5162 (1.2694–1.7630)	0.1259	145.0166	<.0001
AMH [(2.5,5) vs (0,2.5)]	0.6173 (0.3883–0.8464)	0.1168	27.9161	<.0001
AND [≥10 vs (0,5)]	1.0050 (0.7579–1.2520)	0.126	63.5737	<.0001
AND [(5,10) vs (0,5)]	0.4753 (0.2697–0.6810)	0.1049	20.5176	<.0001

**Table 5:** Performance of Model 3.

Measures	Training set	Validation set	Testing set
AUC (95% CI)	0.855 (0.838–0.870)	0.848 (0.791–0.891)	0.846 (0.812–0.875)
Sensitivity (95% CI)	0.362 (0.331–0.395)	0.394 (0.303–0.492)	0.383 (0.324–0.445)
Specificity (95% CI)	0.981 (0.997–0.983)	0.985 (0.974–0.991)	0.981 (0.974–0.986)

CI, confidence interval.

**Figure 2:**
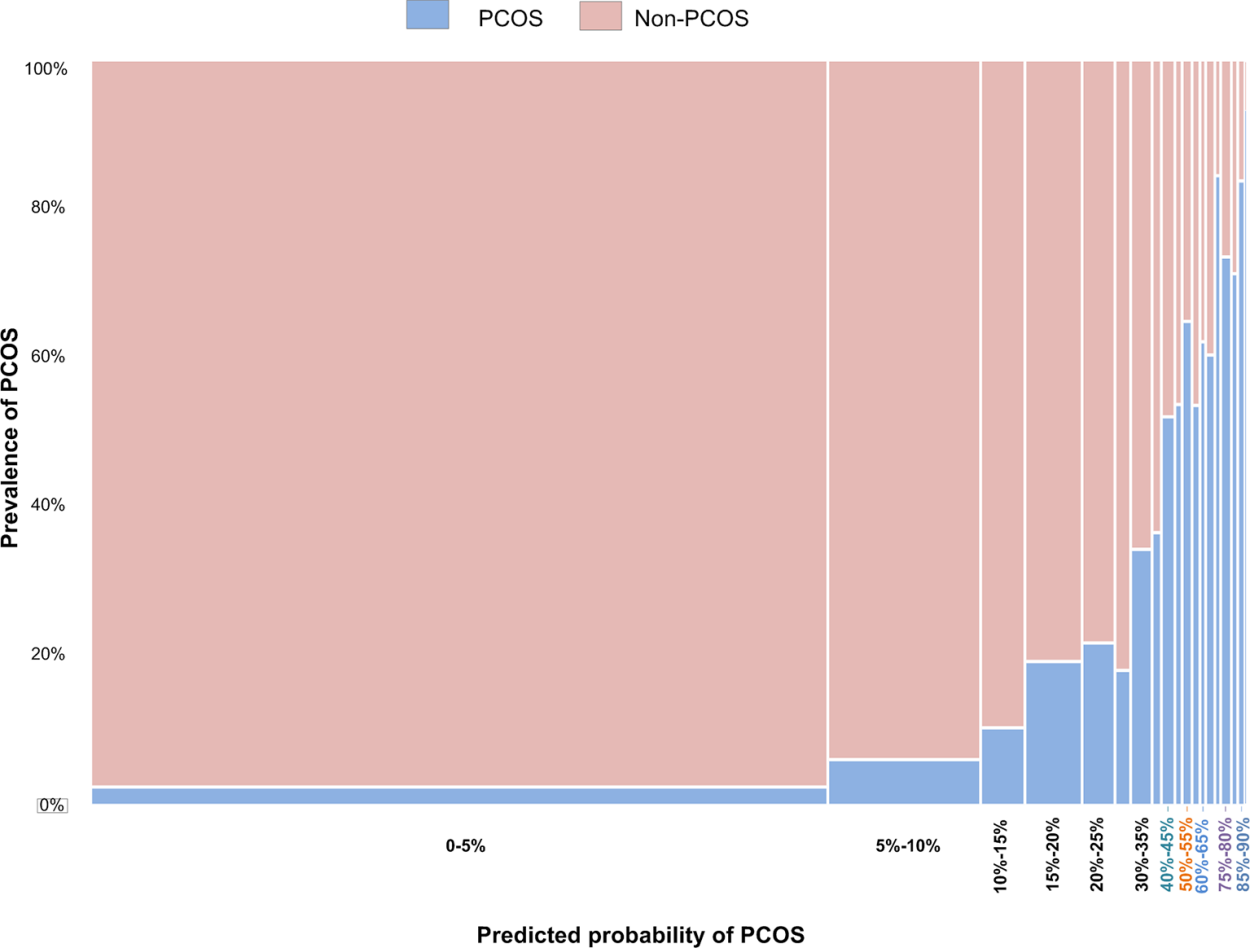
The relationship between the prevalence of PCOS in women being treated by ART and the predicted probabilities using Model 3.

The relationship between predicted probability and the prevalence of PCOS is shown in **Figure 2**. The prevalence of PCOS increased with the predicted probability. **Table 6** shows the top 10 groups of women most highly predicted to have PCOS. The prevalence and predicted probability of PCOS in all groups are indicated in **Supplementary Table 2**.

**Table 6:** The 10 groups of women with highest predicted probability of PCOS.

Group	UML	BMI (kg/m^2^)	AMH (ng/mL)	AND (nmol/L)	Cases of PCOS	Cases of non-PCOS	Incidence of PCOS	Predicted probability of PCOS
	(days)							
1	(60,90)	≥28	≥10	≥10	6	1	85.71%	93.56%
2	>90	≥28	≥10	≥10	8	0	100.00%	93.14%
3	(45,60)	≥28	≥10	≥10	2	1	66.67%	92.56%
4	>90	≥28	(7.5,10)	≥10	2	1	66.67%	92.46%
5	(60,90)	≥28	(7.5,10)	≥10	2	0	100.00%	91.90%
6	>90	≥28	≥10	(5,10)	2	0	100.00%	90.79%
7	>90	≥28	(5,7.5)	≥10	5	2	71.43%	88.01%
8	>90	(24,28)	≥10	≥10	34	3	91.89%	87.69%
9	(35,45)	≥28	(7.5,10)	≥10	2	0	100.00%	87.10%
10	(60,90)	(24,28)	≥10	≥10	20	7	74.07%	87.09%

Our algorithm for diagnosing PCOS has been developed into a website for Chinese ART clinics, (http://121.43.113.123:8888/). In this, the user inputs the required indicators, clicks ‘calculate’, and the results of specific probability of PCOS and risk group of a certain subject are displayed. The grouping criteria are as follows: a low-risk group, with a predicted probability of <10%; a medium-risk group, with a predicted probability of 10% to 50%; and high-risk group, with a predicted probability of >50%.

## Discussion

There are good correlations between AMH level and polycystic ovarian morphology (12–14), and the serum AMH level has been increasingly regarded as a surrogate marker for PCOS and ovarian reserve assessment. Previous studies have found different AMH cut-off values to diagnose PCOS. However, because of small sample sizes, inappropriate controls and heterogeneous AMH assays (15), the application of these cut-offs in the diagnosis of PCOS has been limited. This might be why the introduction of AMH into the diagnosis of PCOS is controversial. We have established a four-item mathematical model (AMH + UML + BMI + AND) instead of a simple AMH cut-off to diagnose PCOS, with AUCs of 0.855, 0.848, 0.846 in the training, validation, and testing sets, respectively. The contributions of each predictor in Model 3 are: AMH 41.2%; UML 35.2%; BMI 4.3%, and AND 3.7%.

Although AMH might serve as a potential diagnostic marker for PCOS, it is not currently recommended as a single-test parameter for this by the International Evidence-based Guideline for the Assessment and Management of Polycystic Ovary Syndrome 2018 (16). Comprehensive models have been established, combining AMH and other indicators (15, 17–19). Vagios et al. (18) have established a diagnostic model using AMH and BMI to predict PCOS. Our model and that one have both revealed the essential roles of AMH and BMI in diagnosing PCOS. Although logistic regression was both used, the differences exist. First, our model did not highlight fixed indicators, but focused on the process of screening predictors from multiple variables. The study by Vagios et al. focused on AMH and BMI in a stratified analysis. Second, our sample size was larger, with cross-validation data and testing data indicating its stability; however, there was no another validation data in the study by Vagios et al., so the diagnostic performance between different study populations is inconclusive. As for the role of age in predicting PCOS, our results shown in **Table 2** indicate that adjusting for UML, serum AMH level, the AFC, BMI, serum AND level and the serum TES level, the contribution of age is small, only 0.2%.

PCOS is mainly a hyperandrogenic disorder, verified in various rodent models using androgen induction (20). However, how excessive androgens are produced remains largely unknown. Studies using mouse models have revealed that AMH is involved in regulating the hypothalamic–pituitary–ovarian axis and might stimulate the production of excess androgens (21, 22). Administration of recombinant human AMH on gestational days 16.5, 17.5 and 18.5 in mice activated the AMH receptor in gonadotropin releasing hormone -secreting neurons and increased the frequency of luteinizing hormone (LH) pulses, leading to increased levels of serum LH and TES and decreased levels of E_2_ and progesterone in pregnant mice on gestational day 19.5 (22). The elevated levels of serum LH and TES induced by high AMH levels lead to oligo-ovulation or anovulation and defective oocyte development in mother mice and their female offspring. Thus, AMH is being increasingly recognized as a potential marker for the diagnosis of such disorders in women undergoing ART.

It has been debated that whether there is a possibility AFC, one of the measures acquired by ultrasonography (9), could be replaced by AMH. Our previous study has shown that AFC can be replaced by AMH in assessing ovarian reserve (23). However, AFC and AMH have different significance. AMH is secreted by immature granulosa cells in a Gn-independent way, while the AFC reflects the small Gn-dependent follicular development. Could AFC be replaced by AMH when screening PCOS? Our results here show that the contribution of AMH to Model 1 (without AFC) is 35.1%, while the contribution of a combination of AMH and AFC in Model 2 (with AFC) is 35.5%, which suggests that AMH could potentially replace AFC in diagnosing or screening PCOS.

The etiology of PCOS is multifactorial (3, 14). Affected patients with or without a normal BMI are observed commonly in our clinical practice. This heterogeneity means that the driving force might differ in patients with PCOS according to their BMI, which is consistent with many other studies (24, 25). Thus, the etiology of such obese patients with PCOS but with normal AMH levels (such as group 96 in **Supplementary Table 2**) might differ from that in normal weight PCOS patients with high AMH levels (such as group 16), so follow-up treatments should also be different.

Because PCOS is so strongly associated with obesity, lean women with PCOS often go undiagnosed for years while they struggle to conceive. In our data, the prevalence of PCOS was 64/1071 (5.98%, CI:4.71%-7.56%) for women with a BMI <18.5 kg/m^2^. When combining this value of BMI<18.5 kg/m^2^ with an AMH level >10 ng/mL, the prevalence of PCOS increased to 21/49 (42.86%, CI:30.02%-56.73%). Similarly, combining the values of BMI <18.5 kg/m^2^ and AMH>10 ng/mL and UML of >90 days, the incidence of PCOS increased to 10/13 (76.92%, CI:49.74%-91.82%). These normal-weight or lean women still face fertility challenges, increased androgen levels and the resulting symptoms (such as acne, hirsutism, and hair loss), and increased risk of diabetes and cardiovascular disease (26). The diagnostic models we have established here might help diagnose these patients in a timely fashion and facilitate their long-term health management.

It has been demonstrated that serum AMH levels in oligomenorrheic girls without evidence of hyperandrogenism are similar to levels in adolescents and adults with PCOS but elevated compared with normal adolescents and adults (27). Combined with the discovery that excessive AMH contributes to ovulatory disorders in mice (21), high AMH levels might be closely associated with oligomenorrhea. In our data, among the first group with a predicted PCOS probability of 93.56%, there are still more than 10% who do not meet the Rotterdam criteria (**Supplementary Table 2**). There is a high probability that these women who have not been diagnosed with PCOS are actually oligomenorrheic without evidence of hyperandrogenism. In addition, the possibility of a missed diagnosis cannot be ruled out.

### Limitations of the Study

First, although our sample size was large, our PCOS diagnostic models are still based on retrospective data, further prospective studies are needed to prove their applicability in ART practice. Second, the different AMH values obtained using diagnostic kits from different manufacturers might also affect the applicability of our models. Third, it has been acknowledged that the measurements of plasma or serum total TES levels in men have adequate sensitivity and clinical utility, but are relatively inaccurate for women because of poor accuracy and sensitivity, which severely limits their clinical utility (28). This might explain why the TES level was not of significance in our models. A TES diagnostic kit with better performance might help to further optimize our diagnostic models. Fourth, other predictors are needed for improving the performance of our models in the future. Finally, our PCOS screening model is established based on data of reproductive aged women from infertility clinics, not adolescent girls, thus is not suitable for PCOS screening in adolescents.

## Conclusion

Women with PCOS are at increased risk of infertility and multiple metabolic symptoms, which need long-term health management. However, the clinical diagnosis of PCOS is complicated, requiring different tests and physical examinations. Currently, screening and diagnosis of PCOS is not an easy task for general gynecologists and primary physicians. Here, we establish an easy applicable PCOS screening model. By entering two serological indicators and the upper limit of the menstrual cycle length and BMI, the probability and the risk of having PCOS could be predicted (http://121.43.113.123:8888/). It can be used to early identify these women who have higher risk of having PCOS, so that they can be further diagnosed, thus contributing to their long-term health management. Moreover, it should be noted that our PCOS screening model is established based on data of reproductive aged women from infertility clinics, not adolescent girls, thus is not suitable for PCOS screening in adolescents.

## Data Availability Statement

The original contributions presented in the study are included in the article/**Supplementary Material**. Further inquiries can be directed to the corresponding author.

## Author Contributions

HX, GF, and KA contributed to manuscript drafting and revising. HX, GF, KA, LC, and YH contributed to data analysis and interpretation. HX, LC, and RY prepared figures and tables. RL and JQ contributed to the conception of the study, manuscript revising and final approval. All authors reviewed the manuscript. All authors contributed to the article and approved the submitted version.

## Funding

This study was supported by the National Natural Science Foundation of China for Distinguished Young Scholars (Grant No. 81925013); the National Key Research and Development Program of China (Grant No. 2018YFC1002104, 2018YFC1002106); the Innovation & Transfer Fund of Peking University Third Hospital (Grant No. BYSYZHZB2020102, BYSYZHKC2021104); the Major National R&D Projects of China (Grant No. 2017ZX09304012-012); National Natural Science Foundation of China (Grant No. 81771650); the Capital Health Research and Development of Special Project (Grant No. 2018-1-4091).

## Conflict of Interest

Author KA is employed by The Predict Health. Author YH is employed by Hangzhou Qingguo Medical Technology Co. Ltd.

The remaining authors declare that the research was conducted in the absence of any commercial or financial relationships that could be construed as a potential conflict of interest.

## Publisher’s Note

All claims expressed in this article are solely those of the authors and do not necessarily represent those of their affiliated organizations, or those of the publisher, the editors and the reviewers. Any product that may be evaluated in this article, or claim that may be made by its manufacturer, is not guaranteed or endorsed by the publisher.
