# Supplementary figures and images for: A Model for Predicting Polycystic Ovary Syndrome Using Serum AMH, Menstrual Cycle Length, Body Mass Index and Serum Androstenedione in Chinese Reproductive Aged Population: A Retrospective Cohort Study
Source: Front Endocrinol (Lausanne). 2022 Mar 17;13:821368. doi: 10.3389/fendo.2022.821368 (PMC8970043; doi:10.3389/fendo.2022.821368)

## Slide 1
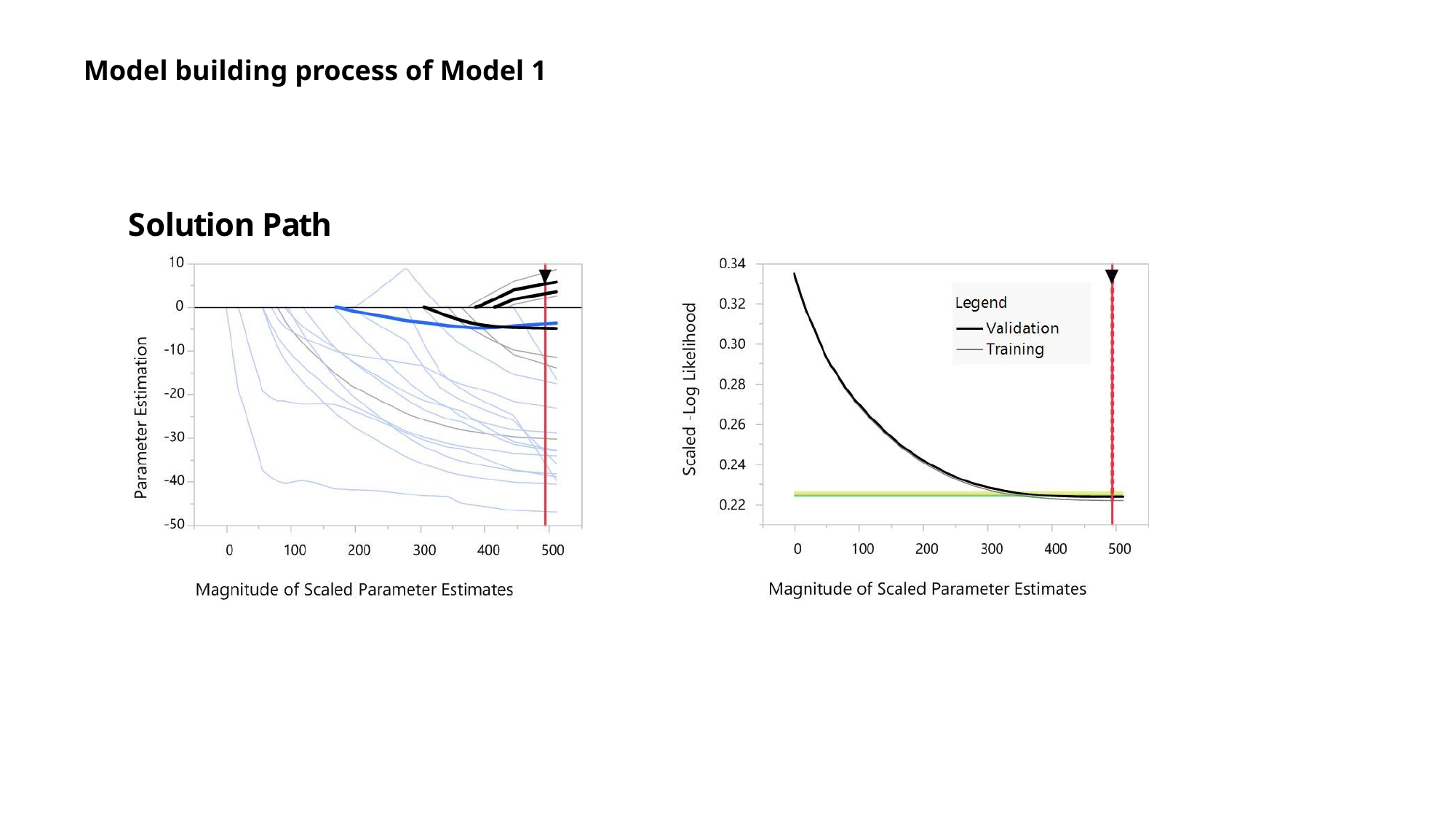

Model building process of Model 1

## Slide 2
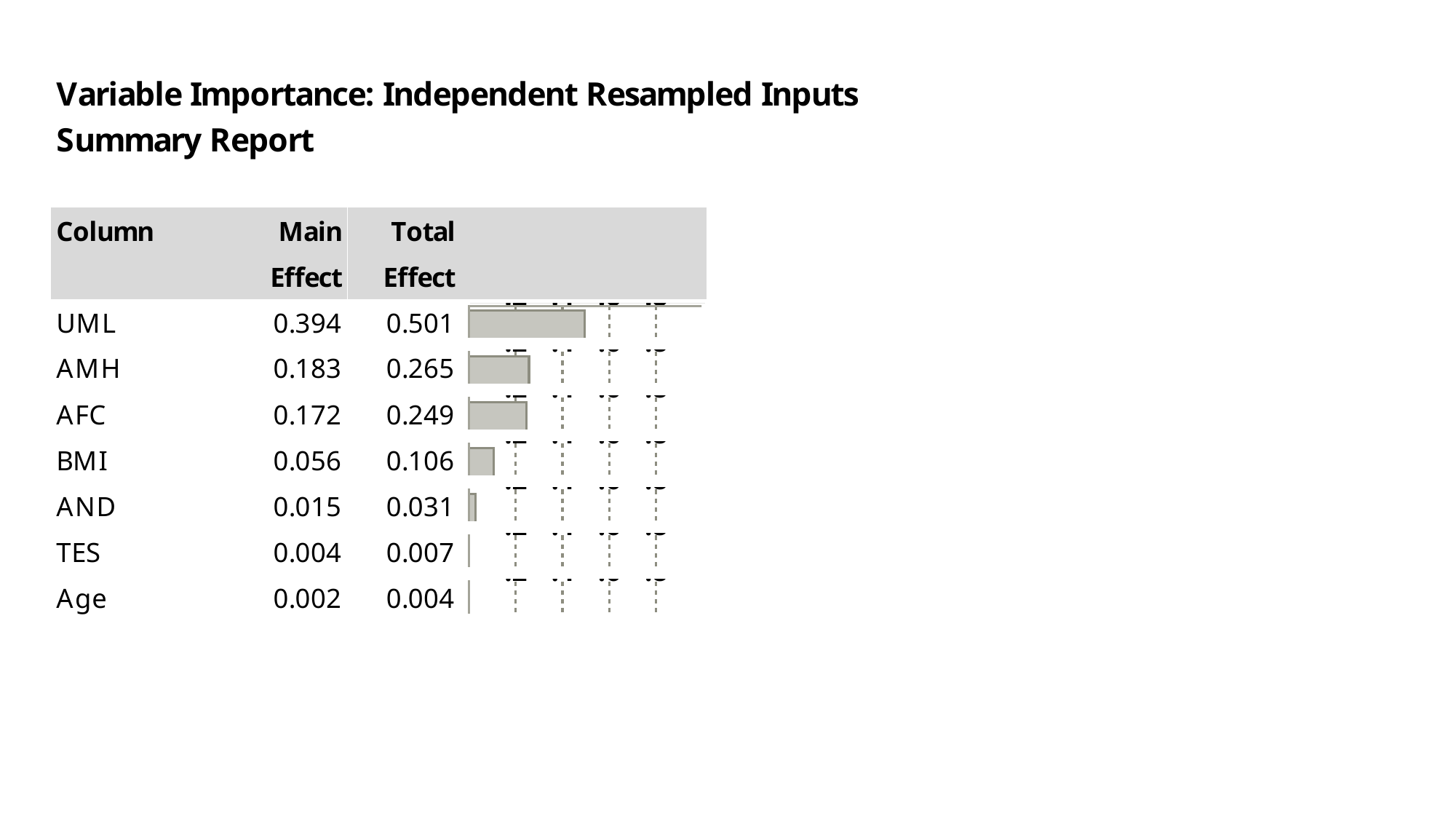

Supplement: Supplementary file 1 [file DataSheet_1.zip › Supplementary Docuement 1_Model building process.PPTX]
